# Carotid Intima-Media Thickness, a Marker of Subclinical Atherosclerosis, and Particulate Air Pollution Exposure: the Meta-Analytical Evidence

**DOI:** 10.1371/journal.pone.0127014

**Published:** 2015-05-13

**Authors:** Eline B. Provost, Narjes Madhloum, Luc Int Panis, Patrick De Boever, Tim S. Nawrot

**Affiliations:** 1 Centre for Environmental Sciences (CMK), Hasselt University, Diepenbeek, Belgium; 2 Environmental Risk and Health, Flemish Institute for Technological Research (VITO), Mol, Belgium; 3 School for Mobility (IMOB), Hasselt University, Diepenbeek, Belgium; 4 Department of Public Health & Primary Care, Leuven University (KU Leuven), Leuven, Belgium; Rutgers University, UNITED STATES

## Abstract

**Introduction:**

Studies on the association between atherosclerosis and long-term exposure to ambient air pollution suggest that carotid intima-media thickness (CIMT), a marker of subclinical atherosclerosis, is positively associated with particulate matter (PM) exposure. However, there is heterogeneity between the different studies concerning the magnitude of this association. We performed a meta-analysis to determine the strength of the association between CIMT and particulate air pollution.

**Methods:**

We queried PubMed citation database and Web of Knowledge up to March 2015 in order to identify studies on CIMT and particulate air pollution. Two investigators selected and computerized all relevant information, independently. Eight of the reviewed epidemiological publications provided sufficient details and met our inclusion criteria. Descriptive and quantitative information was extracted from each selected study. The meta-analysis included 18,349 participants from eight cohorts for the cross-sectional association between CIMT and PM and 7,268 participants from three cohorts for the longitudinal analysis on CIMT progression and PM exposure.

**Results:**

The average exposure to PM_2.5_ in the different study populations ranged from 4.1 to 20.8 µg/m^3^ and CIMT averaged (SD) 0.73 (0.14) mm. We computed a pooled estimate from a random-effects model. In the combined cross-sectional studies, an increase of 5 µg/m^3^ PM_2.5_ was associated with a 1.66% (95% CI: 0.86 to 2.46; *P*<0.0001) thicker CIMT, which corresponds to an average increase of 12.1 µm. None of the studies moved the combined estimate outside the confidence interval of the overall estimate. A funnel plot suggested absence of publication bias. The combined longitudinal estimate showed for each 5 µg/m^3^ higher PM_2.5_ exposure, a 1.04 µm per year (95% CI: 0.01 to 2.07; *P*=0.048) greater CIMT progression.

**Conclusion:**

Our meta-analysis supports the evidence of a positive association between CIMT, a marker of subclinical atherosclerosis, and long-term exposure to particulate air pollution.

## Introduction

Increases in cardiovascular morbidity and mortality have been associated with particulate air pollution levels.[1–4] Altered cardiac autonomic function and atherosclerosis are considered as pathophysiological pathways through which particulate air pollution can influence the cardiovascular system.[5–7] Evidence from animal studies indicates that particulate matter exposure can initiate or accelerate atherosclerosis, substantiating it as a plausible disease causing factor.[8–12]

Carotid intima-media thickness (CIMT) is an important biomarker of subclinical atherosclerosis.[13, 14] Increases in CIMT are associated with both prevalent and incident cardiovascular morbidity and mortality, including coronary heart disease,[15–17] myocardial infarction and stroke.[18] Several epidemiological studies report an association between CIMT and modeled long-term exposure to particulate air pollution.[19–24] However, there is heterogeneity in the effect size of this reported association.

In this current meta-analysis, we determine whether the available observational data, up to March 2015, supports a positive association. Furthermore, we estimate the strength of the association between CIMT and particulate air pollution.

## Methods

### Search strategy and selection criteria

A systematic literature search was performed on PubMed and Web of Knowledge, who were last accessed on 1 March 2015, with no restriction for time of publication. The following search strategies were used: ('particular matter' OR 'air pollution' OR 'PM_10_' OR 'PM_2.5_') AND ('intima media thickness' OR 'carotid intima media thickness' OR 'carotid intima media' OR 'artery intima media thickness' OR 'intima media thickness measurement' OR 'intima media thickness cardiovascular' OR 'carotid intima media thickness measurement' OR 'carotid intima media thickness cardiovascular'). We also considered references found in the literature search. Two investigators (EBP and NM) read all the papers and extracted and computerized the relevant information independently (Tables 1 and 2). This meta-analysis complies with the preferred reporting items of the statement for Meta-analysis Of Observational Studies in Epidemiology (MOOSE).[25]

**Table 1:** Characteristics of the studies included in the meta-analysis of cross-sectional results.

Author	Year	Study	Population	Number of participants	Age, y	Women, %	Exposure	Exposure model	Average PM_2.5_ concentration, μg/m^3^	Average CIMT, mm
Künzli *et al*. [19]	2005	Vitamin E Atherosclerosis Progression Study (VEAPS) and B-Vitamin Atherosclerosis Intervention Trial (BVAIT)	Healthy adults, increased risk of CVD	798	59 ± 10	44	Residential annual mean PM_2.5_	Kriging interpolation based on residential ZIP-code	20.3 ± 2.6	0.76 ± 0.15
Bauer *et al*.[20]	2010	Heinz Nixdorf Recall (HNR) study	General	3,380	60 ± 8	48	Residential annual mean PM_2.5_	Dispersion model in 1 km grids	16.8 ± 1.6	0.66 ± 0.16^a^
Lenters *et al*.[21]	2010	Atherosclerosis Risk in Young Adults study	Young adults	745	28 ± 1	53	Residential annual mean PM_2.5_	Land Use Regression model in 100 m grids	20.7 ± 1.2	0.49 ± 0.05
Tonne *et al*.[22]	2012	Whitehall II study	General	2,347	61 ± 6	34	Residential annual mean PM_10_	Hybrid regression dispersion model based on residential postcode (± 15 addresses)	17.7 ± 1.8^b^	0.79 ± 0.16
Adar *et al*. [23]	2013	Multi-Ethnic Study of Atherosclerosis (MESA)	General	5,276	62 ± 10	52	Residential annual mean PM_2.5_	Complex spatio-temporal based model	16.6 ± 3.7	0.68 ± 0.19
Perez *et al*.[24]	2015	European Study of Cohorts for Air Pollution Effects (ESCAPE) consisting of four cohorts:	n/a	n/a	n/a	n/a	n/a	n/a	n/a	n/a
		IMPROVE-Stockholm	Healthy adults, increased risk of CVD	487	67 ± 0.4	50	Residential annual mean PM_2.5_	Standardized Land Use Regression models of ESCAPE	7.2 ± 1.3	0.85 ± 0.16
		Heinz Nixdorf Recall (HNR) study	General	3,759	60 ± 8	51	Residential annual mean PM_2.5_	Standardized Land Use Regression models of ESCAPE	18.4 ± 1.1	0.68 ± 0.13
		KORA	General	2,646	56 ± 13	52	Residential annual mean PM_2.5_	Standardized Land Use Regression models of ESCAPE	13.6 ± 0.9	0.85 ± 0.14
		Registre Gironi del Cor (REGICOR)	General	2,291	59 ± 12	55	Residential annual mean PM_2.5_	Standardized Land Use Regression models of ESCAPE	14.9 ± 1.6	0.70 ± 0.15

Age, average PM_2.5_ concentration and average Carotid Intima-Media Thickness (CIMT): Values are mean ± SD unless otherwise indicated.

^a^ Values are median ± IQR.

^b^ Average PM_2.5_ concentration calculated based on the assumption that PM_10_ consists for 70% of PM_2.5_.

n/a: not applicable, characteristics are given for each subcohort below.

**Table 2:** Characteristics of the studies included in the meta-analysis of longitudinal results.

Author	Year	Study	Population	Number of participants	Age, y	Women, %	Exposure (model)	Average PM_2.5_ concentration, μg/m^3^	Average CIMT, mm	Average CIMT progression, μm/year
Künzli *et al*.[26]	2010	Vitamin E Atherosclerosis Progression Study (VEAPS), B-Vitamin Atherosclerosis Intervention Trial (BVAIT), Estrogen in the Prevention of Atherosclerosis Trial (EPAT), Troglitazone Atherosclerosis Regression Trial (TART) and Women’s Estrogen-Progestin Lipid-Lowering Hormone Atherosclerosis Regression Trial (WELLHART)	Healthy adults	1,483	59 ± 10	63	Residential annual mean PM_2.5_ (Kriging interpolation)	20.8 ± 2.4	0.78 ± 0.15	2.0 ± 12.9
Adar *et al*. [23]	2013	Multi-Ethnic Study of Atherosclerosis (MESA) study	General	5,276	62 ± 10	52	Residential annual mean PM_2.5_ (spatio-temporal)	16.6 ± 3.7	0.68 ± 0.19	14 ± 53
Gan *et al*.[27]	2014	Multicultural Community Health Assessment Trial (M-CHAT)	General	509	47 ± 9	51	Residential annual mean PM_2.5_ (land-use regression)	4.1 ± 1.5	0.67 ± 0.12	9.2 ± 11.4

Values are mean ± SD

**Fig 1:**
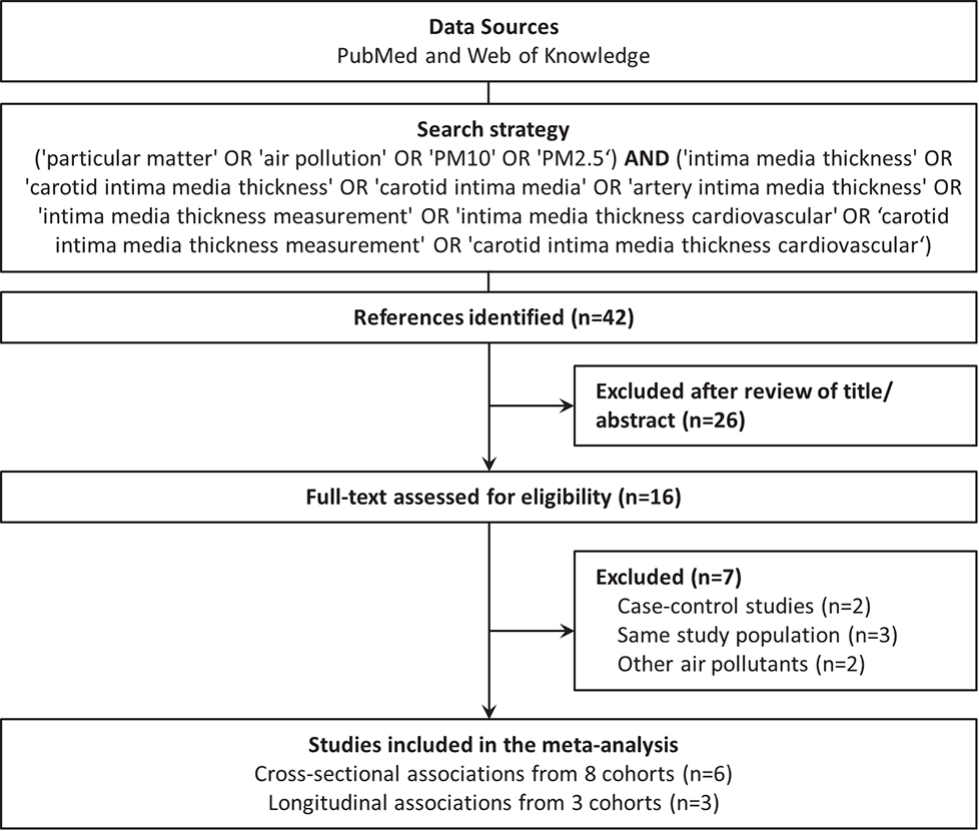
Flow chart of the study selection for meta-analysis.

Selection of the studies was based on the research question, inclusion and exclusion criteria (Fig 1). All studies were reviewed by title and abstract and, if eligible for inclusion, by reading the full text. All types of studies and designs were considered for inclusion. Nevertheless, studies needed to report originally collected data. Therefore, reviews, editorials and debates were excluded.

We selected the studies that used particulate matter with an aerodynamic diameter of 10 μm or less (PM_10_) or 2.5 μm or less (PM_2.5_) as indicators of air pollution. Studies using only other air pollution measures or indicators were excluded. Furthermore, only studies measuring carotid IMT were included.

Out of the 42 initially identified articles, 9 reported a cross-sectional association between CIMT and PM_10_[22] or PM_2.5_[19–21, 23, 24, 28] and 3 reported longitudinal associations between CIMT progression and PM_2.5_[23, 26, 27]. If a group published two or more papers based on the same study population,[23, 28–30] only the publication that provided the most detailed information was included. We selected the results adjusted for gender, age, and BMI as well as for other known correlates of CIMT such as cholesterol levels and smoking status, if provided.

### Statistical analysis

A meta-analytical combined estimate was derived from the point estimate of each separate study weighted by the inverse of the variance (1/SE^2^). In the case that only data for PM_10_ was available (n = 1), we converted the point estimate under the assumption that PM_10_ consist for 70% of PM_2.5_.[31] The combined estimate was computed using a random-effects model and is presented as a percent change in CIMT associated with a 5 μg/m^3^ higher long-term PM_2.5_ exposure for cross-sectional associations. Similarly, we computed a combined estimate based on the studies reporting a longitudinal association between the progression of CIMT and exposure to PM_2.5_. Results of this additional meta-analysis are presented as μm change in CIMT per year for a 5 μg/m^3^ higher long-term PM_2.5_ exposure.

The sensitivity of the cross-sectional findings was examined by recalculating the combined estimate while excluding one study at a time in order to evaluate the influence of individual studies on the combined effect size. If the combined estimate, excluding one study, lies outside the confidence interval of the overall estimate, the excluded study has a disproportionate influence on the combined effect size. Further, between-study heterogeneity was examined using the Cochran Q and I^2^ test. We plotted the association size against the SE of the study in order to investigate publication bias. This should result in a funnel shape (funnel plot) if there is no bias. As an additional sensitivity analysis, we recalculated the combined estimate while using the overall estimate of the European Study of Cohorts for Air Pollution Effects (ESCAPE), replacing the separate estimates. As a final sensitivity analysis, we replaced the point estimates of the overall, between-city, association by the within-city estimate, presented by Adar *et al*.[23]

## Results

### Study selection

Of the 42 studies reviewed, 26 were excluded after review of the title and/or abstract; 19 reported associations with other types of exposures than PM_2.5_ or PM_10_, or another outcome measure than CIMT. Three were reviews, one was on study design, one was performed in an animal model, one reported technical aspects of IMT measurements and one on PM_2.5_ modeling approaches. After assessment of the full-text, 7 additional studies were excluded; three were based on the same study population[28–30], two were case-control studies of which no relevant association size could be computed[32, 33] and 3 reported associations with other air pollution measurements or indicators than PM_2.5_ or PM_10_.[34–36] We identified a set of six studies which investigated the cross-sectional association between CIMT and PM and three longitudinal studies on CIMT progression in association with PM (Fig 1).

### Cross-sectional associations between CIMT and PM

The selection of six cross-sectional studies includes four longitudinal studies investigating baseline cross-sectional associations[20–23], one study reporting baseline cross-sectional analyses of two trials[19] and one reporting cross-sectional results from four different cohort studies within ESCAPE.[24]

The Heinz Nixdorf Recall (HNR) cohort, from the publication by Bauer and colleagues[20], is also one of the cohorts included in ESCAPE.[24] Since different modeling approaches were used for estimating the participants’ exposure to PM_2.5_, differences between the reported point estimates of the two publications are found. We included the point estimates of the most recent publication from ESCAPE by Perez *et al*.[24] in the main meta-analysis and performed a sensitivity analysis using the point estimate from the publication by Bauer *et al*. Therefore, all six studies are listed in chronological order in Table 1.

The five publications included in the main meta-analysis comprised 18,349 participants from 8 cohort studies. The majority of the study populations had an even gender distribution (range: 34 to 55% women) and an average age of 57 years. The average exposure to PM_2.5_ in the different study populations ranged from 7.2 to 20.7 μg/m^3^ and CIMT averaged (SD) 0.73 (0.14) mm.

All studies used modeled PM concentrations based on the participant’s residence averaged over one year prior to the CIMT measurements. Whenever possible, preference was given to mean IMT measurements of the common carotid artery. In all reports, results were adjusted for gender, age and smoking status. Most studies also considered additional covariates including BMI,[20–22, 24] blood pressure[19, 21] and cholesterol levels.[19, 21, 23]

**Fig 2:**
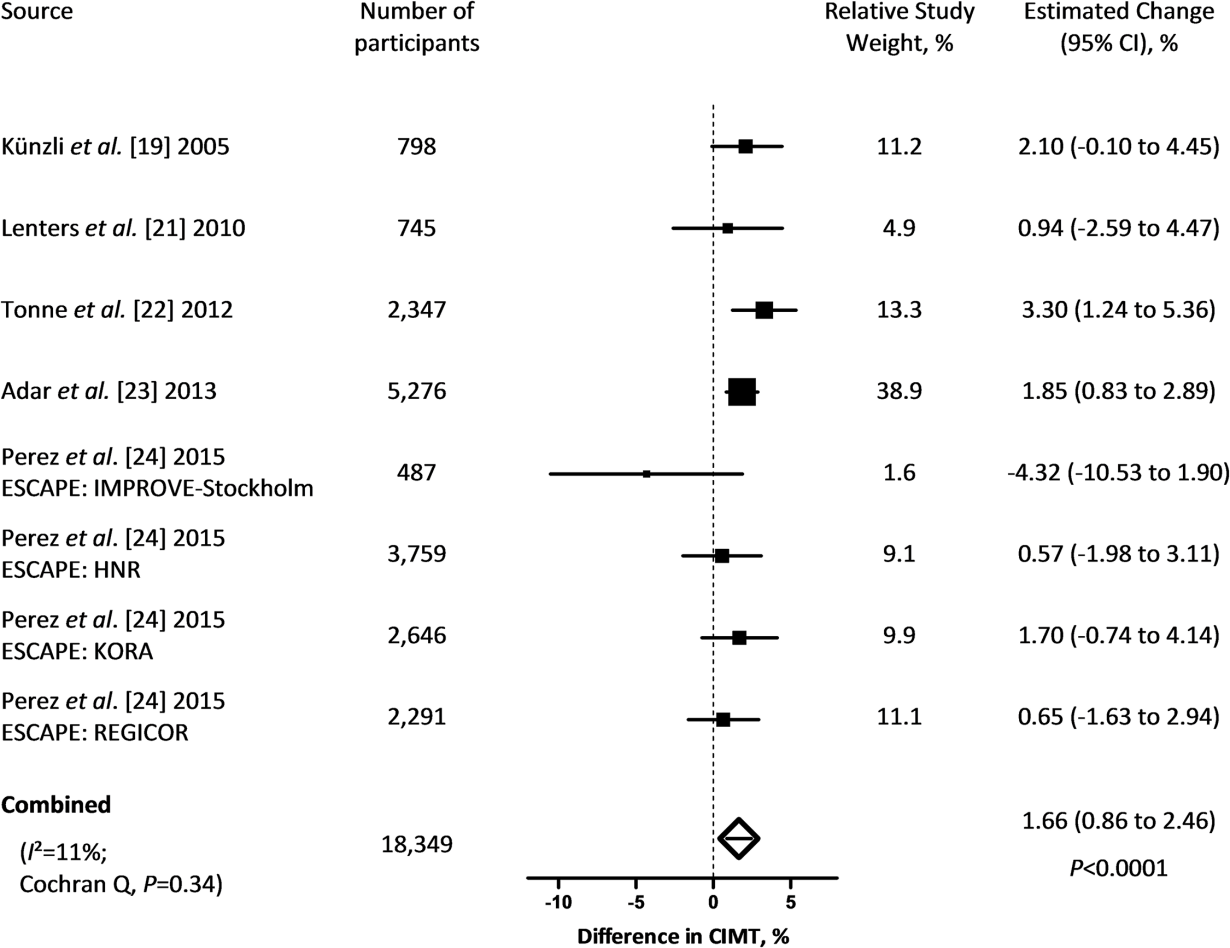
Percent change in CIMT (95% CI) associated with a 5 μg/m^3^ higher long-term exposure to PM_2.5_. Squares represent individual studies. The magnitude of each square represents the inverse of the variance.

**Fig 3:**
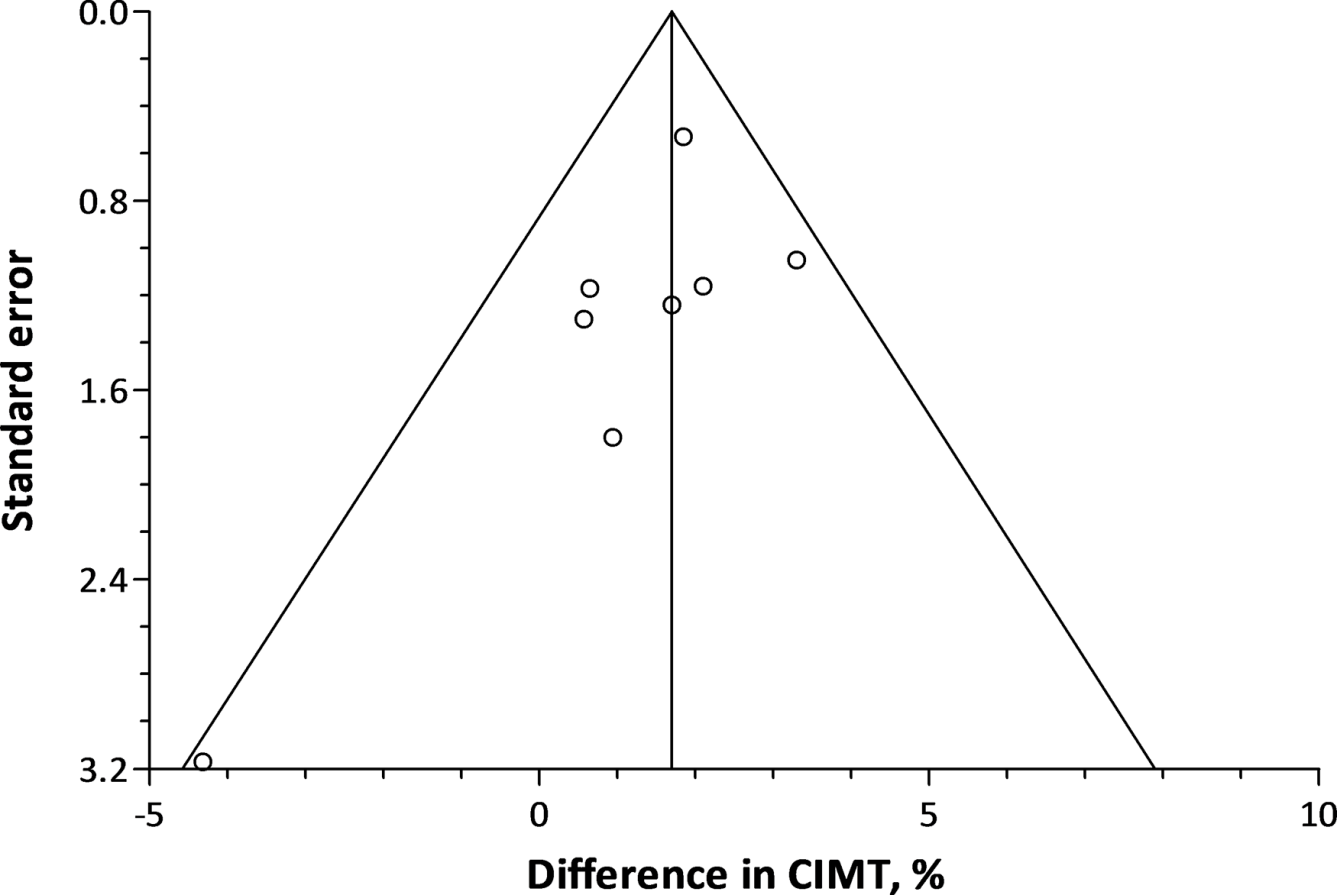
Funnel plot showing the difference in CIMT associated with a 5 μg/m^3^ higher PM_2.5_ exposure against the standard error of each individual cross-sectional study.

The combined estimate showed a 1.66% increment (95% CI: 0.86 to 2.46; *P*<0.0001) in CIMT for each 5 μg/m^3^ higher long-term PM_2.5_ exposure (Fig 2). Cochran Q statistics did not indicate incomparability of the study’s results (*P* = 0.34). Exclusion of Adar *et al*.[23] resulted in a drop in the combined estimate to 1.48% (95% CI: 0.35 to 2.62; *P* = 0.01), whereas it increased to 1.78% (95% CI: 1.07 to 2.49; *P*<0.0001) when omitting the results of the IMPROVE-Stockholm cohort from ESCAPE.[24] None of the studies moved the combined estimate outside the confidence interval of the overall estimate. Including the point estimate of the study by Bauer *et al*.[20] as a result from the HNR study instead of the result from ESCAPE, increased the combined estimate to 1.99% (95% CI: 0.95 to 3.04; *P* = 0.0002). Using the overall point estimate of ESCAPE, instead of the results from the 4 subcohorts, increased the combined estimate to 1.73% (95% CI: 0.92 to 2.54; *P*<0.0001). In a final sensitivity analysis, we replaced the overall, between-city, associations by the within-city associations as reported by Adar *et al*.[23] This lowered the combined estimate to 1.34% (95% CI: 0.30 to 2.38; *P* = 0.01) but the estimate remained within the confidence interval of the overall estimate of the main meta-analysis. The funnel plot did not provide indications of publication bias (Fig 3).

### Progression of CIMT and PM

We identified three longitudinal studies, comprising 7,268 participants, which investigated the association between progression of CIMT and PM_2.5_ of which the characteristics are listed in Table 2.[23, 26, 27] The study populations had an even gender distribution (range: 51 to 63% women) and an average age of 56 years. The average exposure to PM_2.5_ in the different study populations ranged from 4.1 to 20.8 μg/m^3^, CIMT averaged (SD) 0.71 (0.15) mm and average CIMT progression ranged from 2 to 14 μm per year.

**Fig 4:**
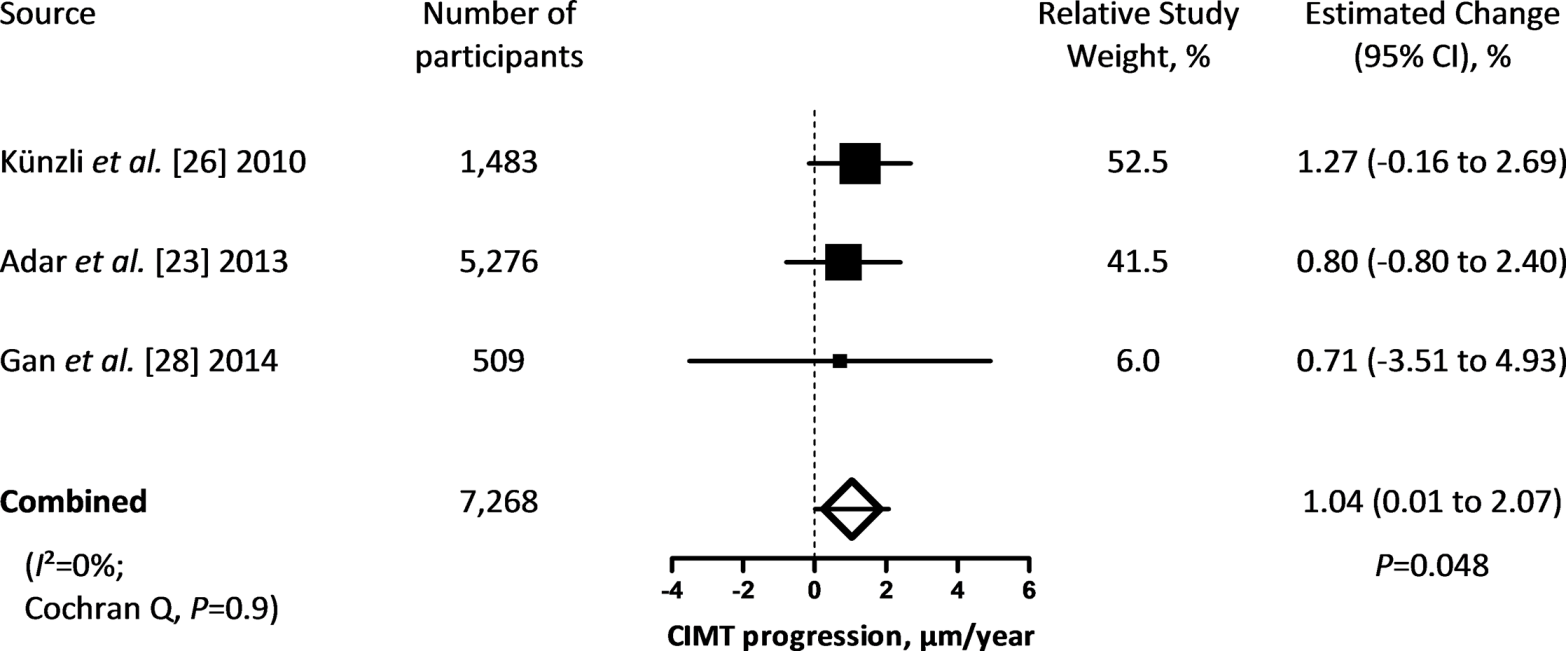
Change in CIMT progression in μm per year (95% CI) associated with a 5 μg/m^3^ higher long-term exposure to PM_2.5_. Squares represent individual studies. The magnitude of each square represents the inverse of the variance.

The combined estimate showed a 1.04 μm per year (95% CI: 0.01 to 2.07; *P* = 0.048) greater CIMT progression for each 5 μg/m^3^ higher long-term PM_2.5_ exposure (Fig 4). Cochran Q statistics did not indicate incomparability of the study’s results (*P* = 0.90). However, when replacing the between-city associations by the within-city associations as reported by Adar *et al*.[23], Cochran Q was significant (*P* = 0.0025) and the random combined estimate changed to 3.64 μm per year (95% CI: -1.21 to 8.50; *P* = 0.14).

## Discussion and Conclusion

The key finding of the present meta-analysis is that IMT of the carotid artery is positively associated with long-term exposure to particulate air pollution. CIMT was 1.66% thicker for a 5 μg/m^3^ increase in PM_2.5_ exposure. These effects were calculated based on cross-sectional results from 8 cohorts comprising 18,349 study participants.

Air pollution is a mixture of several pollutants but epidemiological and lab-based evidence suggests that PM per se might have an important role in the causation of adverse effects.[2] By selecting PM_2.5_ as a common indicator, we envisioned to capture all effects of different sources and components of PM that promotes the pro-atherosclerotic process. Nonetheless, we are limited by the fact that different modeling approaches were used to estimate PM exposure within the different study populations. We expressed the combined estimate for a 5 μg/m^3^ increase, which is realistic. Most urban areas worldwide have PM_2.5_ concentrations greater than the WHO target of 10 μg/m^3^, with a change in the population mean PM_2.5_ exposure of 5 μg/m^3^ needed to match the WHO guidelines set to protect public health. Carotid intima-media thickness is a strong predictor for both prevalent and incident cardiovascular morbidity and mortality, including coronary heart disease, myocardial infarction and stroke.[16–18] If applied to the population at large, our findings have important implications for public health. Based on meta-analytical evidence of prospective studies, each 100 μm increase in CIMT is associated with 8% higher risk of myocardial infarction and 12% higher risk of stroke.[37] Our combined estimate of 1.66% increase in CIMT for a 5 μg/m^3^ increase in PM_2.5_ corresponds to an average increase of 12.1 μm. A reduction in PM_2.5_ of 5 μg/m^3^ in the population at large is therefore likely to result in a 0.94% decreased risk of myocardial infarction and a 1.4% decreased risk of stroke.

Epidemiological studies, such as the ones included in the current meta-analysis, do not prove causation. However, the fact that associations with similar effect size can be observed in different study populations is one of the most important Hill criteria of causation.[38] Furthermore, we performed a meta-analysis on studies reporting longitudinal associations between progression of CIMT and PM exposure, though the number of studies was limited. Results from this meta-analysis suggest that CIMT progression is increased with 1.04 μm per year in association with a 5 μg/m^3^ long-term exposure to PM_2.5_. This further adds to the causality discussion of the findings. In addition to PM, one study on NO_2_[34] and one on black carbon[35] were identified. Both compounds are proxies for traffic-related air pollution. Rivera *et al*.[34] reported a 0.56% (95% CI: -1.5% to 2.6%) thicker CIMT for a 25 μg/m^3^ increase in NO_2_ exposure within the REGICOR study. Wilker and colleagues[35] found that a 1.1% (95% CI: 0.4 to 1.7%) thicker CIMT was associated with a 260 ng/m^3^ higher 1-year average black carbon exposure. Finally, a case-control study in highway toll collectors provided evidence for the effect of traffic-related air pollution on CIMT[32], showing similar effect sizes as a case-control study investigating the effect of biomass fuel smoke exposure on CIMT.[39]

Animal studies show that particulate air pollution can be an underlying cause of the development of atherosclerosis.[8–12] For example, concentrated ultrafine particles caused systemic oxidative stress, an inhibition of the anti-inflammatory capacity of HDL, and larger early atherosclerotic lesions in susceptible Apo lipoprotein E-deficient mice.[9] Oxidative modification of LDL is both a risk factor and a marker of the proatherogenic process.[40, 41] Together with increased blood leukocytes and platelets it can contribute to the initiation and progression of atherosclerosis. Oxidative modification has also been positively associated with individual exposure to air pollution as exemplified by carbon load in lung macrophages of diabetes patients.[42]

The current meta-analysis should be interpreted within the context of its inherent limitations. Meta-analytical evidence might be biased due to the predicament of publication bias and the fact that only studies with positive results are published. However, our funnel plot did not suggest publication bias. Although the number of included publications was small (n = 5), they comprised a large number of participants (n = 18,349) from the general population in different age ranges. Although the magnitude of the association varied between the different studies, our combined estimate was robust and not driven by a single study, as substantiated by the sensitivity analyses.

In conclusion, our results show an overall statistically significant positive association between subclinical atherosclerosis, characterized by carotid intima-media thickness, and long-term exposure to particulate air pollution. Improvement of the air we breathe is a very relevant target to reduce proatherosclerotic effects associated with particulate air pollution in the population.

## Supporting Information

S1 PRISMA Checklist Preferred Reporting Items for Systematic Reviews and Meta-Analyses (PRISMA) checklist. (DOC)

S1 File Full-text excluded articles, with reasons for exclusion. (DOCX)
