# Supplementary material for: Carotid Intima-Media Thickness, a Marker of Subclinical Atherosclerosis, and Particulate Air Pollution Exposure: the Meta-Analytical Evidence
Source: PLoS One. 2015 May 13;10(5):e0127014. doi: 10.1371/journal.pone.0127014 (PMC4430520; doi:10.1371/journal.pone.0127014)
Supplement: S1 File — (DOCX) [file pone.0127014.s002.docx]

**File S1. Full-text excluded articles, with reasons for exclusion.**

The following full-text articles were excluded since they were based on the same study population from the Multi-Ethnic Study on Atherosclerosis (MESA) as Adar et al.[23], which provided the most detailed information.

28. Sun M, Kaufman JD, Kim SY, Larson TV, Gould TR, et al. Particulate matter components and subclinical atherosclerosis: common approaches to estimating exposure in a Multi-Ethnic Study of Atherosclerosis cross-sectional study. Environmental Health. 2013;12(39).

29. Kim SY, Sheppard L, Kaufman JD, Bergen S, Szpiro AA, et al. Individual-level concentrations of fine particulate matter chemical components and subclinical atherosclerosis: a cross-sectional analysis based on 2 advanced exposure prediction models in the multi-ethnic study of atherosclerosis. Am J Epidemiol. 2014;180(7): 718-728.

30. Diez Roux AV, Auchincloss AH, Franklin TG, Raghunathan T, Barr RG, et al. Long-term exposure to ambient particulate matter and prevalence of subclinical atherosclerosis in the Multi-Ethnic Study of Atherosclerosis. Am J Epidemiol. 2008;167(6): 667-675.

The following two case-control studies were excluded since no relevant association size, i.e. percent change in carotid intima-media thickness in association with a 5 µg/m³ higher PM_2.5_ exposure, could be computed.

32. Erdogmus B, Yazici B, Annakkaya AN, Bilgin C, Safak AA, et al. Intima-media thickness of the common carotid artery in highway toll collectors. J Clin Ultrasound. 2006;34(9): 430-433.

33. Tsao TM, Tsai MJ, Wang YN, Lin HL, Wu CF, et al. The health effects of a forest environment on subclinical cardiovascular disease and heath-related quality of life. PLoS One. 2014;9(7): e103231.

The following three publications were excluded since they report associations with other air pollution measures or indicators than PM_2.5_ or PM_10_. Rivera and colleagues report associations with NO_2_, Wilker and colleagues with black carbon and Armijos and colleagues with residential proximity to major roads.

34. Rivera M, Basagana X, Aguilera I, Foraster M, Agis D, et al. Association between long-term exposure to traffic-related air pollution and subclinical atherosclerosis: the REGICOR study. Environ Health Perspect. 2013;121(2): 223-230.

35. Wilker EH, Mittleman MA, Coull BA, Gryparis A, Bots ML, et al. Long-term exposure to black carbon and carotid intima-media thickness: the normative aging study. Environ Health Perspect. 2013;121(9): 1061-1067.

36. Armijos RX, Weigel MM, Myers OB, Li WW, Racines M, et al. Residential exposure to urban traffic is associated with increased carotid intima-media thickness in children. J Environ Public Health. 2015;2015(713540).
